# Diagnostic Use of Skeletal Survey in Suspected Skeletal Dysplasia

**DOI:** 10.4274/jcrpe.v1i6.270

**Published:** 2010-12-08

**Authors:** Amith Kumar Iynapillai Veeramani, Paul Higgins, Sandra Butler, Malcolm Donaldson, Elizabeth Dougan, Roderick Duncan, Victoria Murday, Syes Fasial Ahmed

**Affiliations:** 1 Duncan Guthrie Institute of Medical Genetics, Yorkhill, Glasgow, Scotland; 2 Duncan Guthrie Institute of Medical Genetics, Yorkhill, Glasgow, Scotland; 3 Department of Diagnostic Imaging, Royal Hospital for Sick Children, Yorkhill, Glasgow, Scotland; 4 Department of Orthopaedic Surgery, Royal Hospital for Sick Children, Yorkhill, Glasgow, Scotland; 5 Department of Child Health, University of Glasgow, Yorkhill, Glasgow, Scotland; +00 141 201 05 71+00 141 201 08 37s.f.ahmed@clinmed.gla.ac.ukBone & Endocrine Research Group, Department of Child Health, Royal Hospital for Sick Children, Yorkhill, Glasgow G3 8SJ

**Keywords:** Skeletal dysplasia, diagnosis, skeletal survey

## Abstract

**Objective**: To review the practice of skeletal surveys in cases of suspected skeletal dysplasia.

**Methods**: Retrospective review of records of patients with suspected skeletal dysplasia between December 1997 and December 2005.

**Results**: A diagnosis of a specific skeletal dysplasia was reached in 155 out of a total of 285 suspected cases (54%). In 260 (91%), a record of radiological examination was available and out of these cases, 91 (35%) had a full skeletal survey. A diagnosis was reached in 79% of cases that had a full skeletal survey and in 44% of cases that had a limited survey. A possible skeletal dysplasia was excluded in 44 out of 260 (17%) cases. In 79 out of 260 (30%) cases, skeletal abnormalities were present but a clear diagnosis could not be reached. Over the period of study, there was no clear change in the practice of performing x-rays and the rate of reaching a diagnosis.

**Conclusion**: A clear diagnosis of skeletal dysplasia is not possible in a third of cases and there is a need for greater access to multidisciplinary input.

**Conflict of interest:**None declared.

## INTRODUCTION

Skeletal dysplasia is a general term that refers to abnormal bone and cartilage development. There are almost 400 different kinds of skeletal dysplasias, which can manifest in ways ranging from a barely noticeable abnormality to a severe and lethal condition. Although the exact genetic cause may not be clear in many skeletal dysplasias, tremendous advances have been made over the last decade in the elucidation of the genetic defect in several of these conditions. Whilst the birth prevalence of lethal neonatal short limb skeletal dysplasias may be approximately one in 9000 births (1), population studies report that almost 1 in 2000 newborn infants may have a skeletal dysplasia (2, 3, 4). Although some forms of skeletal dysplasia can be suspected during fetal development, most are usually not identified until after birth when affected children present with skeletal deformity, an increased predisposition to skeletal pain or fractures, or a restriction of linear growth that may affect some parts of the skeleton more than others. In these circumstances, attempts to reach an accurate diagnosis facilitate the provision of reliable information to parents; pave the way for optimal management whilst directing further investigations such as genetic analysis. Whilst the likelihood of reaching a diagnosis has been facilitated by new classifications of skeletal dysplasia by diagnostic groups (5) and molecular pathogenesis (6), improved access to imaging and genetic analysis as well as expert international opinion, it is unclear whether there has been an improvement in the diagnostic yield of cases of suspected skeletal dysplasia. We have, therefore, investigated the use of imaging and its relationship to diagnostic yield in a large contemporary cohort of cases who presented to a children’s hospital for suspected skeletal dysplasia over a period of eight years.

## METHODS

Children who were under the care of the endocrinology, genetics and orthopaedic service at the Royal Hospital For Sick Children, Glasgow between December 1997 and December 2005 and who were clinically suspected to suffer from a skeletal dysplasia were included in the analysis. Cases with increased limb length such as Marfan’s syndrome, contractural arachnodactyly and homocystinuria were excluded. Information from the electronic records, as well as case notes, were retrieved to collect date of birth, year of presentation and details of any radiological imaging that was performed by clinical experts in paediatric radiology. This imaging was referred to as a full skeletal survey, if it consisted of frontal chest, AP pelvis, AP and lateral thoracolumbar spine, lateral skull, AP view of left arm and left hand, and AP view of left leg. If there was unilateral aplasia or hypoplasia of a left-sided limb, then x-rays of the contralateral limb were performed instead. Otherwise, radiological examination restricted to a specific part of the skeleton was termed a limited skeletal survey. Data are presented as medians with 10^th^ and 90^th^ centiles, and differences between groups were assessed using the Wilcoxon signed rank test. The study was approved by the local regional ethics committee as a review of current clinical practice.

## RESULTS

**Skeletal Survey & Diagnostic Yield**

**Figure 1:**
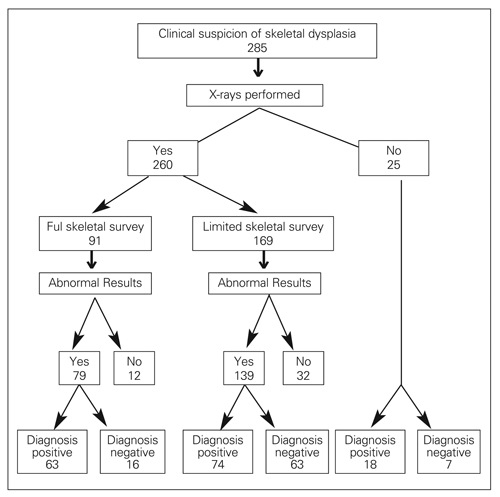
The use of radiological investigations as a diagnostic tool in skeletal dysplasias

Out of 285 cases that presented over the study period, 260 (91%) had a record of radiological examination (Figure 1). Out of these 260 cases, 91 (35%) had a full skeletal survey and the remainder 169 (65%) had a limited radiological examination. In 63 out of 91 (69%) who had a full skeletal survey, a diagnosis of a specific skeletal dysplasia could be reached; 16 (18%) cases remained undiagnosed and in 12 (13%) skeletal dysplasia was excluded. In the 169 cases who had a limited radiological examination, a diagnosis could be reached in 74 (44%); 63 (37%) cases remained undiagnosed and in 32 (19%) cases, skeletal dysplasia was excluded. Amongst the 25 children who did not have any record of radiological examination, a clinical diagnosis had been reached in 18 (72%) and the remainder remained undiagnosed.

**Trends in the Practice of Skeletal Survey & Diagnostic Yield**

**Figure 2A:**
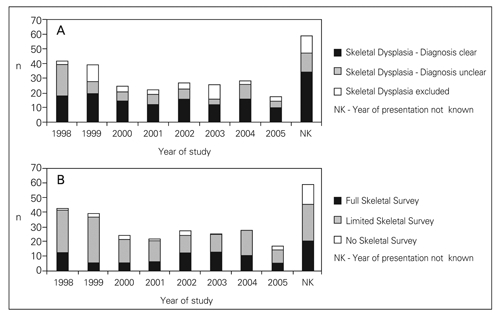
Number of cases per year classified according to (A) diagnostic status or (B) according to whether they had a full skeletal survey, limited skeletal survey or no x-rays

In 226 out of 285 (79%) cases, the year of presentation was clear from the records. The median number of cases of suspected skeletal dysplasia that presented per year was 26 (range, 11-39). The median age at presentation of these 226 cases was 8.9 years (10^th^, 90^th^ centiles; 1.3, 15.4). A diagnosis was reached in 54% of the cases and the likelihood of reaching a diagnosis did not show any change between 1997 and 2005. In approximately 29% of the cases, a skeletal dysplasia was confirmed but a specific diagnosis could not be reached; the percentage of cases in this category between 1997 and 2005 ranged between 15% and 82% (Figure 2A). The median percentage of cases that had a full skeletal survey per year was 31% (range, 15-50) and the median percentage of cases that had a limited skeletal survey per year was 62% (range, 44-79); there was no clear trend in practice over the eight years that were studied (Figure 2B).

**Diagnostic Categories & Skeletal Survey**

Out of the total 285 cases, a clear diagnosis was reached in 155 cases, 86 cases had some form of undiagnosed skeletal dysplasia, and in the remaining 44 cases, the possibility of skeletal dysplasia was dismissed. In 20% of cases of achonlasia (6 out of 28) and osteogenesis imperfecta (7 out of 33) the diagnosis was reached without the help of x-rays (Table 1). The median age at presentation of those cases who had a skeletal survey was 0.75 years (10^th^, 90th centiles; 0.2, 6.8), which was younger than those who were diagnosed without a skeletal survey at 10.8 years (3.9, 15.1) (p=0.01). In addition, it is also notable that there were some conditions, such as metaphyseal dysplasia and hypochonlasia, where a high proportion of cases (9/15 and 7/9, respectively) have had a full skeletal survey for diagnosis. In most cases of skeletal dysplasia, such as fibrous dysplasia, diaphyseal aclasia and hypophosphataemic rickets, a limited skeletal survey was used to aid diagnosis. However, 21% of these cases had a full skeletal survey.

**Genetic Analysis**

A total of 34 cases had a genetic consultation; 15 who had a complete skeletal survey and 19 who had a limited survey proceeded to genetic consultation. Among these 34 cases, genetic analysis was performed in 15 cases; a suspected gene abnormality was identified in 10 cases, and excluded in 2 cases, in 3 cases gene analysis was being considered at the time of this review (Table 1). 

**Table 1:**
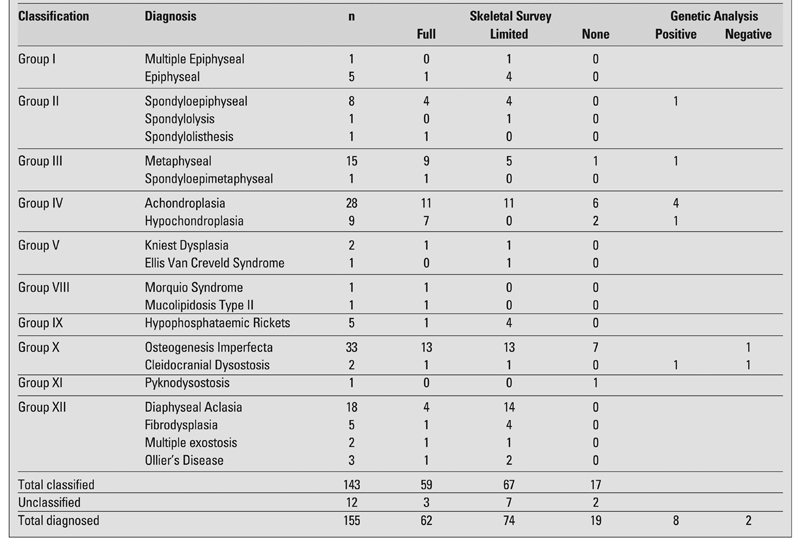
The skeletal dysplasia cases with a clear diagnosis classified according to the Skeletal Dysplasia Research and Teaching Group Classification criteria. The table also shows the number of cases within each diagnostic category who had a full skeletal survey or a limited skeletal survey. In addition, the cases who had genetic analysis and where the results were positive or negative are also highlighted

## DISCUSSION

Between 1997 and 2005, clinicians at Yorkhill, a paediatric tertiary centre encountered approximately 36 cases of suspected skeletal dysplasia each year. Based on physical examination and radiological investigation, approximately 30 cases per year were felt to have a skeletal dysplasia and a clear diagnosis was reached in approximately 20 cases per year. With a reported birth prevalence of 1 in 2000 (2, 3, 4), it is expected that Scotland, which has a birth rate of 55,000 live births per year (7), would have 28 cases of skeletal dysplasia per year, of which approximately 14 would present to the catchment region of our institution. Based on these data, the expected number of cases in the population under the age of 16 years would amount to approximately 220. Between 1997 and 2005, our review identified 241 cases of skeletal dysplasia in patients under the age of 16 years. Although the incidence data as assessed in our current review may be an underestimate as it only represents the experience of a limited number of clinicians, it is probably not too unrealistic, as these clinicians would probably encounter the majority of cases at our institution.

Over the 8 years that were studied, it did not seem that there was a change in the likelihood of reaching a clear diagnosis. In addition, the practice of using skeletal surveys for investigating suspected cases of skeletal dysplasia did not change either. Whether the diagnosis reached was the right one or not and whether there was a change in the level of accuracy of the diagnosis over time was difficult to judge from this review. Whilst a number of guidelines exist in the published and electronic literature for performing radiological analysis in cases of suspected skeletal dysplasia (8, 9, 10, 11, 12), the usefulness of these guidelines in reaching a diagnosis has not been investigated or reported. Published guidelines suggest that the choice between a full or a limited skeletal survey should depend on the presence of disproportion or the presence of specific local skeletal abnormalities (13). Our survey shows that the clinicians’ choice of skeletal survey may depend on the condition suspected. Whilst this may be a useful strategy to employ, it relies on a good knowledge of the skeletal abnormalities that may be expected in that particular condition and how they may be influenced by other factors such as the age of the patient. In fact, some radiographic abnormalities may only become visible at an older age, making serial x-ray evaluation necessary (14). Currently, there are no published data to guide the practice of repeating radiological examination. Although modern x-ray equipment has led to a reduction in radiation dose exposure, the need for targeted and timely skeletal survey remains an important factor that needs further consideration. On the other hand, radiological examination may not be necessary for diagnostic purposes in affected family members with the same phenotype as the index case in whom the diagnosis may have already been confirmed by radiological or genetic analysis. This may apply to familial conditions such as achonlasia, hypochonlasia and osteogenesis imperfecta, as observedalso in our study. 

Whilst radiographic examination plays a significant role in establishing the diagnosis of a skeletal dysplasia, other disciplines such as clinicians, clinical geneticists, molecular biologists and pathologists can also provide important support (15). Advances in clinical and molecular genetics and electronic information exchange have led to an improved understanding as well as tools for managing clinical and radiological information that can facilitate the diagnosis. Our survey showed that clinical genetic involvement was sought only in a relatively small number of cases. It is unclear whether this involvement was sought to confirm the clinically suspected diagnosis by molecular genetic testing, to help with reaching a clinical diagnosis or for genetic counselling. It is likely, however, that a greater involvement of the clinical geneticist with an expertise in this field will lead to a higher diagnostic yield in this area of rapid knowledge advance. 

The observations in this survey have highlighted the need for multidisciplinary input in the diagnosis, as well as management, of skeletal dysplasias and, at our institution, have led to the creation of joint monthly meetings attended by the endocrine, genetics, orthopaedic and radiology teams. It is envisaged that this will provide a multidisciplinary approach to the diagnosis of skeletal dysplasia and lead to the development of future guidelines.

## ACKNOWLEDGEMENT

We are grateful to Mr GC Bennet, Dr R Davidson, Dr. J Tolmie and Dr M Whiteford for case notification.
